# Functional information guided adaptive radiation therapy

**DOI:** 10.3389/fonc.2023.1251937

**Published:** 2024-01-04

**Authors:** R. Craig Herndon

**Affiliations:** Hillman Cancer Center, Radiation Oncology, University of Pittsburgh Medical Center, Williamsport, PA, United States

**Keywords:** precision medicine, information theory, biomarkers, bioinformatics, radiomics, complexity

## Abstract

**Introduction:**

Functional informaton is introduced as the mechanism to adapt cancer therapies uniquely to individual patients based on changes defined by qualified tumor biomarkers.

**Methods:**

To demonstrate the methodology, a tumor volume biomarker model, characterized by a tumor volume reduction rate coefficient, is used to adapt a tumor cell survival bioresponse radiotherapy model in terms of therapeutic radiation dose. Tumor volume, acquired from imaging data, serves as a surrogate measurement for tumor cell death, but the biomarker model derived from this data cannot be used to calculate the radiation dose absorbed by the target tumor. However, functional information does provide a mathematical connection between the tumor volume biomarker model and the tumor cell survival bioresponse model by quantifying both data sets in the units of information, thus creating an analytic conduit from bioresponse to biomarker.

**Results:**

The information guided process for individualized dose adaptations using information values acquired from the tumor cell survival bioresponse model and the tumor volume biomarker model are presented in detailed form by flowchart and tabular data. Clinical data are used to generate a presentation that assists investigator application of the information guided methodology to adaptive cancer therapy research.

**Conclusions:**

Information guided adaptation of bioresponse using surrogate data is extensible across multiple research fields because functional information mathematically connects disparate bioresponse and biomarker data sets. Thus, functional information offers adaptive cancer therapy by mathematically connecting immunotherapy, chemotherapy, and radiotherapy cancer treatment processes to implement individualized treatment plans.

## 1 Introduction

Cancer therapies produce biological responses (bioresponses) that exchange functional information with surrogate biological markers (biomarkers) (1). A method leveraging this information exchange between paired bioresponse-biomarker processes to create individualized cancer therapies was introduced. The bioresponse model characterizes tumor cell death in terms of the therapeutic dose required to achieve the desired effect, whereas the tumor volume biomarker model serves as a surrogate for tumor cell death. The goal was to use biomarker model data to quantify dosimetric changes in cancer therapy using the bioresponse model.

Functional information-guided adaptations of patient cancer treatments were presented using a radiation therapy bioresponse model and a tumor volume (TV) biomarker model based on imaging data. System bioresponse was quantified using a linear quadratic exponential (LQ) cell survival model common to radiotherapy, *S* = *f*(*D*) = *f*(*n · d*), where *S* is tumor cell survival, *D* is the prescribed cumulative dose, *n* is the number of treatments or fractions (the first fraction is administered on day zero, i.e., *n* = 0), and *d* is the fractional treatment dose (2). Fractionation, *n*, of radiation treatment doses, *d*, was designed to optimize the balance between sterilizing cancerous cells and minimizing damage to healthy tissues (3, 4). A TV model, *V* = *g*(*n* · *r*), illustrates how tumor death, defined by a tumor volume reduction rate coefficient, *r*, in fractions, *n*, is directly related to tumor cell survival because it is a quantitative imaging biomarker (QIB), i.e., it is a good measure of tumor anatomical characteristics acquired from medical imaging data when used correctly (5, 6). This biomarker model, *V* = *g*(*n* · *r*), is used to illustrate how information on the current biological status of the patient’s tumor is used, along with information from the cancer model, *S* = *f*(*n · d*), to adapt the patient’s treatment dose, *d*, to their current biological status.

Adaptive radiation therapy (ART) has been an active area of research for more than 25 years (7–22). An NRG Oncology review of the current state of ART defined ART as patient radiation treatments that are individualized based on changes in patient weight, tumor and organ geometry, and biological response (bioresponse), as well as stochastic variations such as organ deformation, filling change, and respiration and peristaltic motion (23). Changes in tumor location and morphology are quantified using imaging technologies, and the necessary dosimetric changes are made offline or online (23). Information-guided ART complements these current expansive efforts in precision medicine research and clinical implementation by mathematically linking biologically complex tumor responses to their qualified tumor biological markers (biomarkers), thereby offering individualization of patient treatments based on their biological profiles (1). Information-guided adaptive therapy is based on the premise that the effect measured by a biomarker is mathematically linked to the therapeutic cause. Modeling the series of biological complexities that connect qualified bioresponse/biomarker relationships has not yet been realized. However, information transmission through this bioresponse/biomarker relationship can be mathematically modeled and leveraged to adapt to therapeutic treatments. The bioresponse and biomarker models used in this study were intended to demonstrate real-world applications. The selected models were based on the current radiobiological knowledge and clinically available technologies. However, the premier of functional information as a mechanism that mathematically connects complex processes that were previously unconnected is the focus of this study. The intent was to clearly demonstrate the method in a step-by-step manner to make the application readily reproducible by interested investigators.

## 2 Materials and methods

### 2.1 Functional information

The radiation dose, *d*, acting upon a biological system causes a bioresponse that is modeled in terms of cell survival, *S* = *f*(*n · d*). Because the cell survival model, *S*, cannot be directly measured, a valid surrogate or biomarker model, *V*, is used to obtain an indirect quantification of the system response, where *V* = *g*(*n · r*) (24, 25). Both the TV biomarker model, *V = g*(*n · r*), and the cell survival bioresponse model, *S = f*(*n · d*), provide information regarding how much of the population survives at a specific dose. Functional information, *I_f_* (Equation 1), quantifies the value of the number of information-carrying real-valued decimal digits (dits) associated with the datasets derived from the cell survival bioresponse model, *S* = *f*(*n · d*), and TV biomarker model, *V* = *g*(*n · r*) (1). Functional information from the TV biomarker data, *I_V_*, and functional information from the cell survival model, *I_S_*, both quantify the relative cancer-cell-killing effectiveness of radiotherapy in units of information (dits). Functional information, *I_f_*, being the average of individual data information components, $i_{j}=-\text{log}(f{\left(x_{j}\right)}_{N})$ , serves as the basis for adaptation of bioresponses using biomarkers, where *m* is the number of equiprobable measurements or data points, and the subscript *N* means that the model data have been normalized to ensure $0\leq f{\left(x\right)}_{N}\leq 1$ across all data points, *x*.


(1) $$I_{f}=\frac{1}{m}\sum_{j=0}^{m}i_{j}=-\frac{1}{m}\sum_{j=0}^{m}\log\left(f{\left(x_{j}\right)}_{N}\right)$$

Next, overviews of the cell survival bioresponse model, *S*, the TV biomarker model, *V*, and the information-guided adaptation formula are presented. Information transmission through a qualified bioresponse–biomarker (*S*–*V*) relationship forms the mathematical basis for the adaptive radiation therapy formula using *I_S_* and *I_V_* information values.

### 2.2 Cell survival bioresponse model

The bioresponse model used in this radiotherapy application was the tumor cell survival model. Cell survival models are used in oncology because they provide information regarding how much of the tumor cell population survives a specific dose of radiation, cytotoxic drugs, or another cell-killing agent (4). Tumor cell survival, *S*, under radiotherapy stress is described by the linear-quadratic (LQ) model (Equation 2), where tumor cell survival is a function of *n* well-separated fractions of radiation dose, *d* (Gy), and alpha (*α*) and beta (*β*) are parameters describing cell radiosensitivity (2, 26). Proliferation of tumor cells during radiotherapy treatment is modeled by the term containing the total number of days of treatment, *T*, the tumor cell repopulation starting time, *T_k_*, and the tumor cell doubling time, *T_p_*. Physicians prescribed a total treatment dose, *D* = *n* · *d*, for each patient which was defined by a schedule of *n* treatments each at dose, *d* (Gy). The radiation therapy prescribed treatment schedule used in this application is 28 fractions, *n* = 28, with a dose, *d*, of 1.8 Gy per fraction for a total dose, *D*, of 50.4 Gy. The LQ cancer cell survival model is used in this application because it is widely used in the clinic due to its biologically relevant and mathematically straight forward interpretation of the effects of clinical fractionation of dose (27, 28). The LQ cell survival data were converted into information, *I_S_*, to determine a value quantifying the relative tumor cell-killing effectiveness of the radiation dose in units of information (dits).


(2) $$S=f\left(n,d\right)=\exp\left(-nd\left(\alpha +\beta d\right)+\text{ln}\left(2\right)(T-T_{\text{k}}\right)/T_{\text{p}})$$

### 2.3 Tumor volume biomarker model

A quantitative imaging biomarker (QIB) is an objectively measured characteristic derived from an *in vivo* image as an indicator of normal biological processes, pathogenic processes, or response to therapeutic intervention (24). Tumor volume is recognized as a QIB by the quantitative imaging biomarkers alliance (QIBA) (29). Quantitative imaging biomarkers are becoming clinically relevant by comprehensively standardizing the technical aspects of image acquisition, analysis algorithms, processes, and clinical validation (30). Tumor volumes derived from magnetic resonance (MR) images are strong QIB candidates because their superior soft tissue resolution enhances quantitative estimates of tumor volume, disease extent, and burden (31, 32). Since changing tumor volume, *V*, provides a method for determining the response to radiotherapy, it serves as a QIB for tumor death and is used to assess the effectiveness of radiotherapeutic treatment (33).

**Figure 1:**
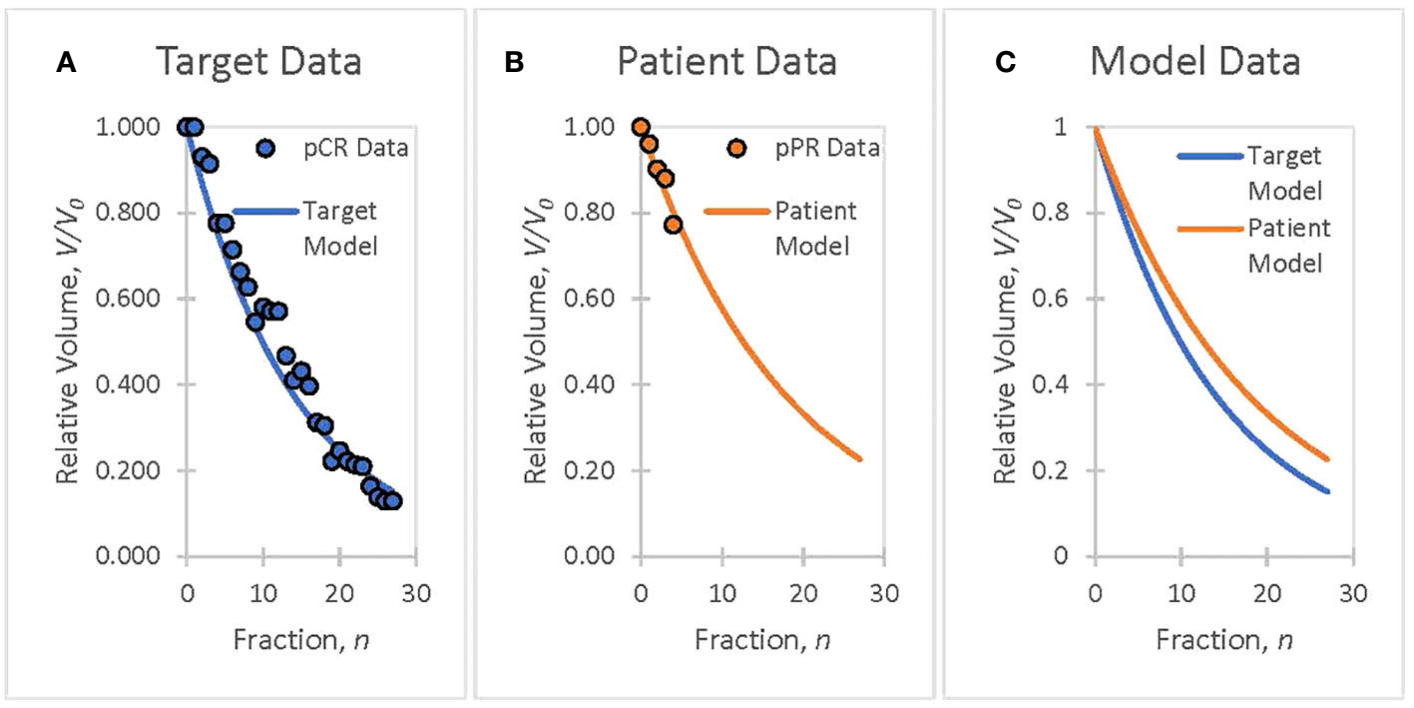
The goal is to ensure that patient treatment responses match target responses using patient biomarker information, analyzed offline or online, and to adapt therapeutic doses to ensure that patient treatments track the planned course of action. **(A)** Clinical data from rectal cancer patients with a pathological complete response (pCR) are used to create a TV biomarker model, *V* = *g*(*n · r*), with a volume reduction rate of *r* = 0.070. **(B)** Week 1 data from rectal cancer patients with a pathological partial response (pPR) are used to create a TV biomarker model, *V* = *g*(*n,r*), with a volume reduction rate of *r* = 0.055. **(C)** The target model (pCR Fit), selected as representative of a successful treatment course, serves as the goal for adaptive treatment. The patient model (pPR fit) will serve to demonstrate the adaptation of a patient’s treatment to a targeted treatment (*Section 3*).


Tumor volume data collected by Bostel et al. from eight patients with locally advanced rectal adenocarcinoma were used to illustrate the methodology (34). Rectal cancer patient tumor volumes, *V*, measured from MR T2-weighted imaging data are well modeled by a linear exponential function, *V* = *g(n,r) = V*_0_exp(−*rn*) in (Equation 3), where *V*_0_ is the initial tumor volume, *n* is the fraction number, and *r* is the TV reduction rate coefficient corresponding to a specific radiotherapy dosimetry schedule. All patients received 50.4 Gy in 28 fractions and concurrent chemotherapy with 5-fluoruracil (300 mg/m^2^ body surface area daily). Clinical data from four patients with pathological complete response (pCR) were used as the basis of the TV model (**Figure 1A**). Pathological partial response (pPR) data from four patients’ first treatment weeks were used to create a patient TV model (**Figure 1B**) and to demonstrate the adaptation methodology in *Section 3*.

(3) $$V=V_{0}\cdot g\left(n,r\right)=V_{0}\cdot \exp\left(-r\cdot n\right)$$

Tumor volume is converted to information, *I_V_*, and, like cell survival information, *I_S_*, offers a description of how tumor cells are killed by radiation dose in units of information (dits). The biomarker response information, *I_V_*, along with the bioresponse information, *I_S_*, were used to determine the bioresponse model parameter-of-interest, radiation dose, *d*. The biomarker model (Equation 3) is based on clinical data and serves the purpose of illustrating the application of adapting patient radiotherapy treatments using each individual patient’s biological data. Analysis of different radiation therapy treatment schedules, the utility of different tumor biomarkers, tumor pathology, and staging all belong to future research that describes clinical implementation (35).

### 2.4 Information guided dose adaptation

Functional information is the mathematical mechanism that allows the adaptation of prescribed radiation therapy using tumor volume measurements. The formula used to relate bioresponse, *I_S_*, and biomarker information, *I_V_*, and ultimately adapt the patient dose using their current biomarker status is determined using information about the prescribed state of the therapy and the adapted state of the therapy (1). The exponential bioresponse (Equation 2), and biomarker (Equation 3) models both have prescribed (*S* and *V*) and adapted (*S’* and *V’*) states, which correspond to the prescribed information (*I_S_* and *I_V_*) and adapted information (*I_S'_* and *I_V'_*). The functional information derived from the tumor volume exponential-based model was linearly mapped from the prescribed model to the adapted model, *I_V'_* = *k_V_I_V_*. The corresponding functional information derived from the cell survival exponential-based model is also defined linearly and equivalently as *I_S'_* = *k_S_I_S_*, such that *k_V_* = *k_S_* = *k* (1). Functional information allows for the creation of an adaptation formula (Equation 4), defined from the bioresponse–biomarker ratio, *I_S'_*/*I_V'_*, and permits the calculation of informational changes due to parametric changes implemented in the exponential-based models to achieve an adaptive target goal expressed in the adapted bioresponse model *S’*.


(4) $$I_{S^{\prime }}=I_{S}\frac{I_{V^{\prime }}}{I_{V}}$$

Depending on the complexity of the bioresponse and biomarker models and the changes made to adapt the models to a target goal, *k* could be a function, not a coefficient. However, functional information generated by the exponential models used in this study is linearly related, and *k* becomes an adaptation coefficient that specifies the fractional change in both the information defined by the investigator’s adaptive goal for the biomarker and the corresponding fractional change in the bioresponse information (1). The fractional change in information, *k*, between the prescribed and adapted biomarker information defines the fractional change, *k*, between the prescribed and adapted bioresponse information because they describe the same biological system. Given these models, the investigator’s goal, defined by the fractional change in biomarker information, is implemented in the fractional change in bioresponse information. The adaptation formula (Equation 4) is elegantly simple because functional information presents a linear relationship that mathematically connects the nonlinear disparate bioresponse and biomarker models.

When a physician prescribes a therapeutic radiation dose, *D* = *n · d*, for a patient, a bioresponse dataset is derived from the cell survival model, *S*, based upon their prescription. The tumor volume, *V*, biomarker data set, corresponding to the prescription, is then derived using measured data collected at each fraction during the treatment course, so that the physician can determine whether the current prescription outcome is satisfactory or if the prescription needs to be adapted to the measured outcome. If adaptation is desired, the parameters in the tumor volume model are adjusted to meet the target goal. Next, the adapted bioresponse information, *I_S'_*, is determined using Equation (4), which is then used to determine the adapted dose, *d’*, necessary to adapt the bioresponse to the desired goal (see *Section 3*). The information-adaptation formula in Equation (4) provides a way to adapt each individual patient treatment plan using their current biology by linearly connecting two nonlinear models, *S* and *V*.

## 3 Results

**Figure 2:**
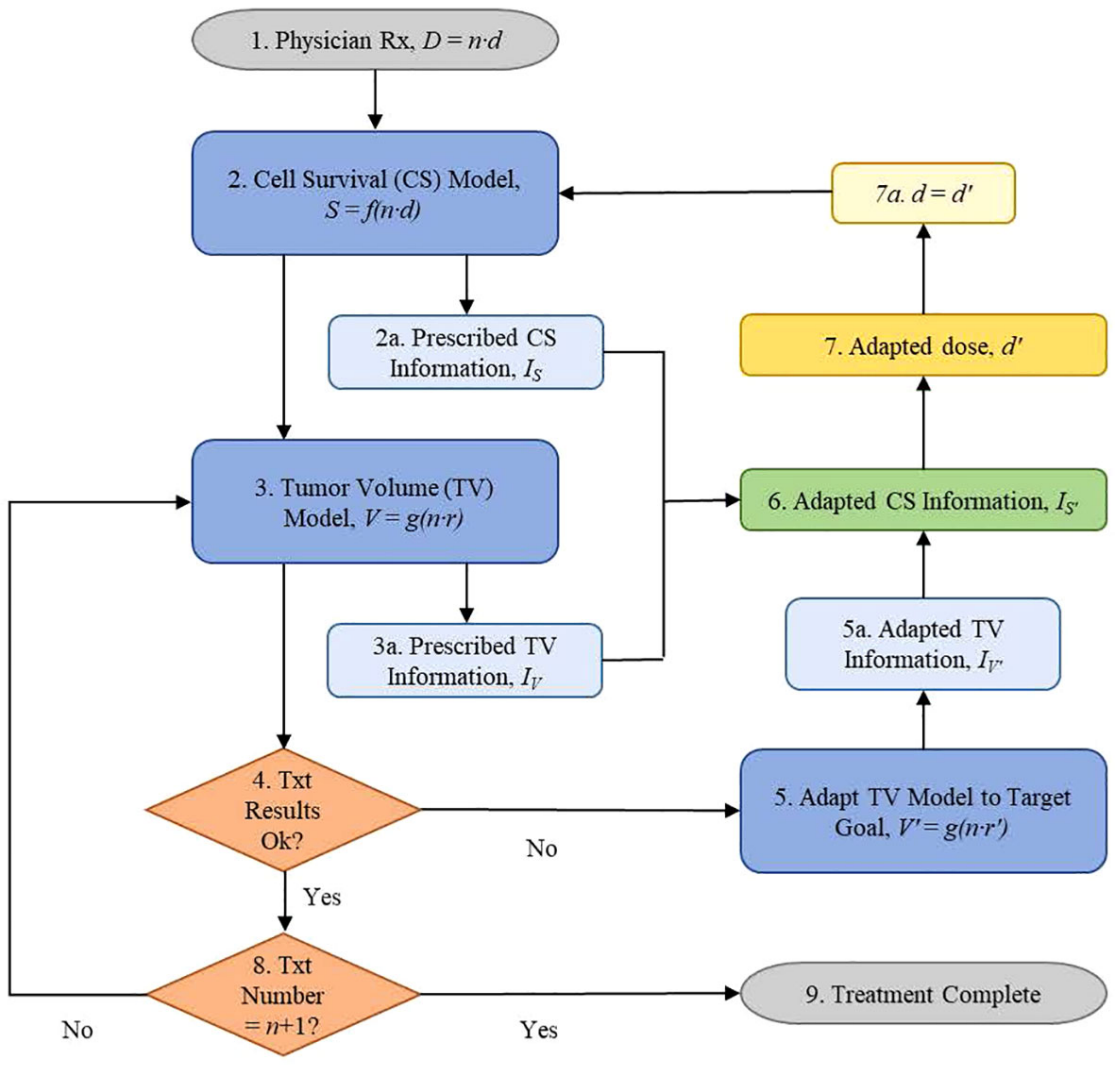
Information-guided radiation therapy dose adaptation flowchart of the steps followed by a treating physician using information to adapt patient radiotherapy treatments via quantitative dosimetric changes based on their current biological status.

Clinical data were presented to illustrate the functional information-guided methodology applied to adaptive radiation therapy. Tumor volumes (TV) measured from T2 MR images, acquired from four neoadjuvant rectal cancer patients who had pathological partial responses (pPR) to treatment were used to simulate the treatment response of a patient during their first week of treatment (**Figure 1B**). Tumor response, as quantified by the TV reduction rate (*r* = 0.055) from the patient TV model, was not considered to be sufficiently aggressive when compared to the target TV model (**Figure 1C**) with a TV reduction rate, *r’* = 0.070. The decision is made to adapt the current prescription, *D* = 50.4 Gy = 28 · 1.8 Gy, by increasing the fraction dose, *d*, to achieve the response defined by the target TV model. The application of the information-guided radiation therapy dose adaptation methodology to this example is outlined in the flowchart in **Figure 2**. Cell survival and tumor volume data were based on clinical data (26, 34).

Flowchart Step 1 (**Figure 2**): The physician prescribes the total dose, *D*, and treatment schedule, *n* · *d*. For example: *D* = *n* · *d* = 50.4 Gy, where the dose, *d*, for each treatment is 1.8 Gy and the number of fractions, *n*, is 28.

Flowchart Step 2: The cell survival data, *S*, were derived using Equation (2). Example: Using *α* = 0.3 (Gy)^−1^ and *β* = 0.03 (Gy)^−2^, *T* = 38 d, *T_k_* = 21 d, *T_p_* = 10 d, the prescription cell survival information is determined to be I_S_ = 3.7359 dits (**Table 1**) using Equation (1) (**Figure 2** Step 2a). The first fraction was obtained on day zero, *n* = 0.

**Table 1:** Prescribed tumor cell survival information, I_S_, derived using the tumor cell survival, *S*, bioresponse model (Equation 2), and functional information formula (Equation 1).

Fraction Number	Fraction Day, *n*	Cell Survival, *S*	Normalized Cell Survival	${\text{i}}_{\text{S}}$ (dits)	${\text{I}}_{\text{S}}$ (dits)
1	0	3.249E+00	1.000E+00	0.0000	3.7359
2	1	1.718E+00	5.288E−01	0.2767	
3	2	9.084E−01	2.796E−01	0.5535	
4	3	4.803E−01	1.478E−01	0.8302	
5	4	2.540E−01	7.818E−02	1.1069	
6	5	1.343E−01	4.134E−02	1.3837	
7	6	7.102E−02	2.186E−02	1.6604	
8	7	3.755E−02	1.156E−02	1.9371	
9	8	1.986E−02	6.111E−03	2.2139	
10	9	1.050E−02	3.232E−03	2.4906	
11	10	5.552E−03	1.709E−03	2.7673	
12	11	2.936E−03	9.035E−04	3.0441	
13	12	1.552E−03	4.778E−04	3.3208	
14	13	8.208E−04	2.526E−04	3.5975	
15	14	4.340E−04	1.336E−04	3.8743	
16	15	2.295E−04	7.063E−05	4.1510	
17	16	1.213E−04	3.735E−05	4.4277	
18	17	6.417E−05	1.975E−05	4.7045	
19	18	3.393E−05	1.044E−05	4.9812	
20	19	1.794E−05	5.522E−06	5.2579	
21	20	9.486E−06	2.920E−06	5.5346	
22	21	5.016E−06	1.544E−06	5.8114	
23	22	2.652E−06	8.164E−07	6.0881	
24	23	1.403E−06	4.317E−07	6.3648	
25	24	7.416E−07	2.283E−07	6.6416	
26	25	3.921E−07	1.207E−07	6.9183	
27	26	2.074E−07	6.382E−08	7.1950	
28	27	1.096E−07	3.375E−08	7.4718	

Refer to Flowchart Step 2. The first dose was administered on day zero (n = 0).

Flowchart Step 3: Biomarker data are derived using the measured data (34) and Equation (3). Example: Normalized tumor volumes, based on week 1 data from Bostel et al., had a TV reduction rate coefficient of *r* = 0.055 (**Figure 1B**). These data were used to model the patient tumor volume response to the prescribed treatment. Information for the prescribed TV model is calculated, ${\text{I}}_{\text{V}}=0.3225$ dits in **Table 2** using Equation (1) (**Figure 2** Step 3a).

**Table 2:** Prescribed tumor volume information, ${\text{I}}_{\text{V}}$ , derived using the tumor volume, *V*, biomarker model (Equation 3), and the functional information formula (Equation 1).

Fraction Number	Fraction Day, *n*	Week 1 Relative Tumor Volume, *V*	Patient Tumor Volume Model	${\text{i}}_{\text{V}}$ (dits)	${\text{I}}_{\text{V}}$ (dits)
1	0	1.0000	1.0000	0.000E+00	0.3225
2	1	0.9610	0.9465	2.389E−02	
3	2	0.9020	0.8958	4.777E−02	
4	3	0.8800	0.8479	7.166E−02	
5	4	0.7720	0.8025	9.554E−02	
6	5	–	0.7596	1.194E−01	
7	6	–	0.7189	1.433E−01	
8	7	–	0.6805	1.672E−01	
9	8	–	0.6440	1.911E−01	
10	9	–	0.6096	2.150E−01	
11	10	–	0.5769	2.389E−01	
12	11	–	0.5461	2.627E−01	
13	12	–	0.5169	2.866E−01	
14	13	–	0.4892	3.105E−01	
15	14	–	0.4630	3.344E−01	
16	15	–	0.4382	3.583E−01	
17	16	–	0.4148	3.822E−01	
18	17	–	0.3926	4.061E−01	
19	18	–	0.3716	4.300E−01	
20	19	–	0.3517	4.538E−01	
21	20	–	0.3329	4.777E−01	
22	21	–	0.3151	5.016E−01	
23	22	–	0.2982	5.255E−01	
24	23	–	0.2822	5.494E−01	
25	24	–	0.2671	5.733E−01	
26	25	–	0.2528	5.972E−01	
27	26	–	0.2393	6.210E−01	
28	27	–	0.2265	6.449E−01	

Refer to Flowchart Step 3. The first dose was administered on day zero (n = 0).

Flowchart Step 4: If the tumor-killing effectiveness of the current radiation dose, *d*, is acceptable, then the treatment continues with the previously adapted dose. Otherwise, the physician will modify the treatment schedule based on the biomarker results. Example: Week 1 data produced a patient model with a low TV reduction rate coefficient, *r* = 0.055, compared with the target model with a TV reduction rate coefficient, *r’* = 0.070 (**Figure 1C**). Treatment results are not satisfactory; therefore, the decision is to pursue a more aggressive treatment by escalating the treatment dose, *d* (Step 5).

Flowchart Step 5: Target TV data, fit from Bostel et al. (**Figure 1A**), served as the adaptation goal. The data were derived using Equation (3) (*r’* = 0.070) and the functional information formula of Equation (1), respectively (**Table 3**). Example: Adapted patient biomarker information increases from the prescribed biomarker information value, ${\text{I}}_{\text{V}}=0.3225$ dits, to the adapted biomarker information value, ${\text{I}}_{V^{\prime }}=0.4104$ dits (**Table 3**) (**Figure 2** Step 5a).

**Table 3:** Adapted tumor volume information, ${\text{I}}_{V^{\prime }}$ , derived using the adapted tumor volume, *V’*, biomarker model (Equation 3) and the functional information formula (Equation 1).

Fraction Number	Fraction Day, *n*	Relative Tumor Volume, *V’* (cc)	Target Tumor Volume Model	${\text{i}}_{V^{\prime }}$ (dits)	${\text{I}}_{V^{\prime }}$ (dits)
1	0	1.0000	1.0000	0.000E+00	0.4104
2	1	1.0000	0.9324	3.040E−02	
3	2	0.9300	0.8694	6.080E−02	
4	3	0.9140	0.8106	9.120E−02	
5	4	0.7760	0.7558	1.216E−01	
6	5	0.7760	0.7047	1.520E−01	
7	6	0.7140	0.6570	1.824E−01	
8	7	0.6630	0.6126	2.128E−01	
9	8	0.6270	0.5712	2.432E−01	
10	9	0.5460	0.5326	2.736E−01	
11	10	0.5810	0.4966	3.040E−01	
12	11	0.5710	0.4630	3.344E−01	
13	12	0.5710	0.4317	3.648E−01	
14	13	0.4670	0.4025	3.952E−01	
15	14	0.4100	0.3753	4.256E−01	
16	15	0.4320	0.3499	4.560E−01	
17	16	0.3970	0.3263	4.864E−01	
18	17	0.3120	0.3042	5.168E−01	
19	18	0.3050	0.2837	5.472E−01	
20	19	0.2220	0.2645	5.776E−01	
21	20	0.2460	0.2466	6.080E−01	
22	21	0.2220	0.2299	6.384E−01	
23	22	0.2140	0.2144	6.688E−01	
24	23	0.2090	0.1999	6.992E−01	
25	24	0.1640	0.1864	7.296E−01	
26	25	0.1380	0.1738	7.600E−01	
27	26	0.1290	0.1620	7.904E−01	
28	27	0.1290	0.1511	8.208E−01	

Refer to Flowchart Step 5. The first dose was administered on day zero (n = 0).

Flowchart Step 6: Adapted patient cell survival functional information was calculated using Equation (4). Example: Adapted cell survival information is calculated, $I_{S^{\prime }}=4.7548$ dits, to accommodate the target goal set in step 4 (*r’* = 0.070). This calculation requires the tumor’s prescribed cell survival information, *I_S_*, the prescribed TV information *I_V_*, and the adapted TV information ${\text{I}}_{V^{\prime }}$ (**Figure 2**, steps 2a, 3a, and 5a).

Flowchart Step 7: The adapted dose, *d’*, is the dose that produces the adapted cell survival information value, ${\text{I}}_{S^{\prime }}$ determined in step 6. This is accomplished through an iterative process using (Equations 1-4), where *d’* is increased until the target ${\text{I}}_{S^{\prime }}$ is achieved. Example: Using the adapted information value from Step 6 as the target, $I_{S^{\prime }}=4.7548$ dits, the patient’s daily prescribed treatment dose, *d* = 1.8 Gy, is iteratively increased to, *d’* = 2.2134 Gy, until the adapted information value, ${\text{I}}_{S^{\prime }}$ (**Table 4**), equals the calculated information, $I_{S^{\prime }}=4.7548$ (Equation 4). The adapted dose becomes the updated prescription dose, *d* = *d’* (step 7a), and the adaptive treatment cycle continues (**Figure 2**, step 2).

**Table 4:** Adapted tumor cell survival information, ${\text{I}}_{S^{\prime }}$ , derived using the tumor cell survival bioresponse model, *S’*, and the adapted radiotherapy dose (*d’* = 2.2134 Gy) determined iteratively using Equations (1)-(4) (see Flowchart Step 7).

Fraction Number	Fraction Day, *n*	Cell Survival, *S’*	Normalized Cell Survival	${\text{i}}_{\text{S"}}$ (dits)	${\text{I}}_{\text{S"}}$ (dits)
1	0	3.249E+00	1.000E+00	0.0000	4.7548
2	1	1.444E+00	4.444E−01	0.3522	
3	2	6.417E−01	1.975E−01	0.7044	
4	3	2.852E−01	8.777E−02	1.0566	
5	4	1.267E−01	3.901E−02	1.4088	
6	5	5.632E−02	1.734E−02	1.7611	
7	6	2.503E−02	7.704E−03	2.1133	
8	7	1.112E−02	3.424E−03	2.4655	
9	8	4.944E−03	1.522E−03	2.8177	
10	9	2.197E−03	6.763E−04	3.1699	
11	10	9.764E−04	3.005E−04	3.5221	
12	11	4.339E−04	1.336E−04	3.8743	
13	12	1.929E−04	5.936E−05	4.2265	
14	13	8.571E−05	2.638E−05	4.5787	
15	14	3.809E−05	1.172E−05	4.9309	
16	15	1.693E−05	5.210E−06	5.2832	
17	16	7.523E−06	2.315E−06	5.6354	
18	17	3.343E−06	1.029E−06	5.9876	
19	18	1.486E−06	4.573E−07	6.3398	
20	19	6.603E−07	2.032E−07	6.6920	
21	20	2.935E−07	9.032E−08	7.0442	
22	21	1.304E−07	4.014E−08	7.3964	
23	22	5.796E−08	1.784E−08	7.7486	
24	23	2.576E−08	7.928E−09	8.1008	
25	24	1.145E−08	3.523E−09	8.4530	
26	25	5.087E−09	1.566E−09	8.8053	
27	26	2.261E−09	6.959E−10	9.1575	
28	27	1.005E−09	3.093E−10	9.5097	

The first dose was administered on day zero (n = 0).

Flowchart Step 8: Continue treatment until the patient receives the prescribed number of treatments.

Flowchart Step 9: Treatment is completed when the course schedule is finished.

## 4 Discussion

The linear exponential tumor volume (TV) model fits the data well and offers the simplicity of a single-rate coefficient to quantify TV reduction, *r*. The TV model offers no specific mechanistic insight beyond the global parameter *r*; however, it offers a model based on quality data that can be easily adapted. A multiparametric mechanistic TV model is the goal; however, it is not required to generate useful information for treatment adaptation. If the investigators are convinced that the tumor volume data are reliable, then any exponential-based curve fit is suitable for adaptation. The linearity in the adaptation formula, $I_{V^{\prime }}=k_{V}I_{V}$ , is maintained because the prescribed TV model, *V*, and adapted TV model, *V’*, are exponential-based functions.

Adjusting the TV reduction rate coefficient (*r* = 0.055 → *r’* = 0.070, see Flowchart) to achieve an adaptation goal is a logical approach because exponential differences are readily apparent to the investigator (see **Figure 1C**). However, the adapted biomarker information, $I_{V^{\prime }}=k_{V}I_{V}$ , can be directly calculated (bypassing **Figure 2** step 5) by choosing an appropriate adaptive constant, *k_V_*. The selection of an appropriate adaptive constant that reflects the physician’s adaptive goal becomes more evident with accumulated experience. Adaptation using the adaptive constant *k_V_* instead of the TV reduction rate coefficient *r* is an option that ensures the validity of the adaptive formula (Equation 4) by maintaining the criterion $I_{V^{\prime }}=k_{V}I_{V}$ , upon which the formula is based.

The functional information-guided adaptive formula (Equation 4), as outlined in this study, is a linear analytical tool for nonlinear systems. The application of this formula requires that the values of the bioresponse information, *I_S_*, and biomarker information, *I_V_*, are comparable. This requirement is met when a qualified bioresponse–biomarker pair is used. Expansion of the exponential-based LQ and TV models to incorporate more biological parameters related to tumor death will not change the linear relationship of Equation (4). For example, by replacing the single broadly defined TV reduction coefficient *r* of Equation (3) with multiple exponential parameters associated with tumor death measured at the macroscopic scale (vascularity, hypoxia, heterogeneity, etc.), would not alter the linear relationship of Equation (4).

Daily tumor volume monitoring allows for the ongoing adaptation of the TV reduction rate coefficient to accommodate the unique biological complexities associated with each individual patient. Tumor volume biomarker information was adapted in the Results section by changing the TV reduction rate coefficient from the measured *r* = 0.055 to the target *r’* = 0.070. Implementing this change would increase the fractional dose to the tumor from *d* = 1.8 Gy to *d* = 2.1 Gy with a corresponding total dose, *D*, increase from the original prescription of 50.4 Gy to 59.6 Gy. This dose escalation is acceptable if ongoing treatment monitoring continually adjusts the prescription schedule and prevents excessive doses to organs at risk while optimizing the dose to the tumor. This adaptation response uses the radiation dose, *d*, in the LQ model as the adaptation parameter of interest, fractionation, *n*, or both dose and fractionation could be adjusted to adapt the treatment schedule.

The ability to use information from quality-measured data to adjust model data has potential applications in other radiobiological models and opens research opportunities for personalizing tumor control and normal tissue complication models. The information workflow defined in Equation (4) could be used to correlate patient tumor α/β ratios with treatment schedules and pathology, leading to prescriptions based on individual biological profiles. This workflow presents opportunities for investigators to fine-tune the effectiveness of different combinations of omic parameters in their models.

Adaptive radiation therapy is currently implemented in clinics using technology that incorporates sophisticated medical imaging and artificial intelligence (AI) capabilities to expedite decision-making processes to help locate and identify targets and adjust dosimetry based on anatomical changes (23, 36–38). There is a trade-off between treatment effectiveness, measured by successful outcomes, and treatment efficiency, measured by human and economic costs. However, AI promises to improve both its effectiveness and efficiency (39). Currently, the costs associated with ART include increased patient treatment times and the requirement for state-of-the-art equipment and specialized personnel. Information guidance, as demonstrated in the *Results* section, will be integrated into the existing ART environment using pretreatment, during-treatment, and post-treatment data in both offline and online analysis formats. The goal is to combine information guidance with AI to produce individualized ART and improve clinical outcomes, thereby fulfilling the promise of increased effectiveness and efficiency.

Information from comparable bioresponse–biomarker pairs await application to imaging biomarkers beyond tumor volume, including FDG PET/CT as an imaging biomarker for cancer treatment under the specified conditions listed in the QIBA Profile (40). Other applications include quantitative MRI biomarkers such as tumor cell density and mobile protein content, which are higher in aggressive tumors and tumor hypoxia (32, 41). Real-time treatment adaptations using this information-based methodology are now conceivable owing to technological advances that present real-time imaging data (23, 41, 42). This information-guided methodology complements current ART research efforts by adjusting dosimetry based on applicable biological profiles.

The adaptation formula is applicable to all biomarker data, including molecular profiles obtained from patient biospecimens. Combining biospecimens and imaging biomarkers will present a wide range of information-guided applications beyond radiotherapy, including systemic cancer treatments such as chemotherapy and immunotherapy (43, 44). Biomarkers associated with cancer processes, such as the TV biomarker used in this study, will be part of ongoing research and development efforts to formally discover, validate, and qualify biomarkers (32, 43, 45). The discovery, validation, and qualification processes require categorization of expertise from multiple disciplines. For example, comparing biomarkers of interest from combined chemotherapy and radiotherapy patient data, such as that used in this study, to cohorts using radiotherapy alone requires radiation biology expertise to identify if and what comparisons are justified. This information data qualification effort, positioned at the hub of multiple disciplines, would help develop an information database used to analyze individual patient responses and augment current efforts to generalize adaptive radiation therapy to adaptive cancer therapy.

## 5 Conclusions

The application of functional information to radiotherapy solves the previously intractable problem of quantitatively linking bioresponse models to biomarker models and offers the implementation of individualized cancer treatment plans based on biological profiles. Additional research avenues are available when this information is used in conjunction with other oncological research areas, such as chemotherapy and immunotherapy.

## Data availability statement

The original contributions presented in the study are included in the article/supplementary material. Further inquiries can be directed to the corresponding author.

## Author contributions

The author confirms being the sole contributor of this work and has approved it for publication.
